# The Effect of Different Extraction Techniques on the Bioactive Characteristics of Dill (*Anethum graveolens*) Essential Oil

**DOI:** 10.1002/fsn3.70089

**Published:** 2025-03-24

**Authors:** Ambreen Fatima, Muhammad Adnan Ayub, Nasrin Choobkar, Muhammad Zubair, Kim D. Thomspon, Amjad Hussain

**Affiliations:** ^1^ Department of Chemistry University of Sahiwal Sahiwal Pakistan; ^2^ Plant Biotechnology Research Center, Kermanshah Branch Islamic Azad University Kermanshah Iran; ^3^ Department of Chemistry, Faculty of Science University of Gujrat Gujrat Pakistan; ^4^ Aquaculture Research Group Moredun Research Institute Penicuik UK; ^5^ Institute of Chemistry University of Okara Okara Punjab Pakistan

**Keywords:** antimicrobial activity, DPPH scavenging, essential oil, GC–MS, superheated steam distillation, Total antioxidant capacity (FRAP)

## Abstract

This study explores the extraction of *Anethum graveolens* essential oil (EO) using advanced techniques, including Superheated Steam Extraction (SHSE), and compares them with traditional methods such as Hydro Distillation (HD), Steam Distillation (SD), and Supercritical Carbon Dioxide Extraction (SCF‐CO_2_). The novelty of this research lies in the comprehensive evaluation of SHSE, a relatively underexplored method, for its effectiveness in enhancing both yield and biological activities of the EO. This study provides a detailed comparative analysis of antioxidant and antimicrobial properties across different extraction methods. The SHSE method yielded essential oil with the highest antioxidant activity, including DPPH scavenging (87.48%) and total antioxidant capacity (measured by FRAP, 163.06 mg/L), outperforming traditional methods. The EO's high content of key components such as carvone, limonene, and dillapiole significantly contributes to its enhanced biological activities. These findings underscore the superior efficacy of SHSE in extracting essential oils, offering new insights into their potential applications in health and wellness, which set this study apart from previous work.

## Graphical abstract

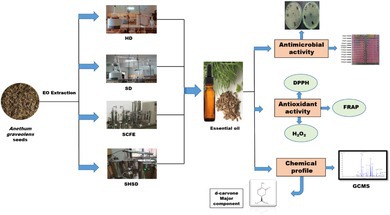

**Abbreviations**
ATCC: American Type Culture Collection
BHT: Butylated Hydroxytoluene
CFU: colony‐forming units
DMSO: dimethyl sulfoxide
DPPH: 2,2‐diphenyl‐1‐picrylhydrazyl
DPPH‐FRSA: DPPH‐free radical scavenging activity
EO: essential oil
GC–MS: gas chromatography–mass spectrometry
H_2_O_2_
: hydrogen peroxide
HD: hydrodistillation
MIC: minimum inhibitory concentration
RPA: reducing power ability
RSM: response surface methodology
SC‐CO_2_
: supercritical carbon dioxide extraction
SCF‐CO_2_
: supercritical fluid carbon dioxide
SCWE: subcritical water extraction
SD: steam distillation
SHSE: superheated steam extraction

## 1 Introduction

Foodborne diseases, caused by a variety of parasites, microbes, and viruses, are a major concern to human health (Patra and Baek 2016). Foodborne pathogens tend to be introduced during food packaging and distribution. They secrete toxic substances, leading to food deterioration and spoilage, and can proliferate within the packaged food. The primary pathogens responsible include *Escherichia coli* , *Staphylococcus aureus* , pathogenic *Vibrio* spp., and *Campylobacter jejuni* . Preservatives, synthetic or natural compounds, are commonly added to inhibit microbial growth. Natural preservatives are preferred over synthetic ones due to their effectiveness and minimal adverse effects. Essential oils are natural preservatives due to their antibacterial, antifungal, and antiviral properties (Ju et al. 2019), as well as their antioxidant activities (Pezantes‐Orellana et al. 2024).

Antioxidants are substances that inhibit oxidation in materials, even when used in minimal quantities. These compounds counteract free radicals that play a role in disease development. By mitigating the damaging impacts of free radicals, antioxidants help decrease the incidence of diseases associated with oxidative stress (Ayub, Hanif, et al. 2023; Konfo et al. 2023). Essential oils comprising eugenol, carvacrol, and thymol as their major constituents can preserve lipids, carbohydrates, proteins, and organic molecules due to their antioxidant activity (Amorati et al. 2013; Żukowska and Durczyńska 2024). In recent years, there has been a growing interest in using natural sources of antioxidants derived from plants owing to their low toxicity at minimal concentrations (Ayub, Hanif, et al. 2023). Additionally, essential oils can extend the shelf life of baked goods and meat products due to their inhibitory effects on spoilage bacteria (Khaleque et al. 2016; Saeed et al. 2022). In addition to their antioxidant properties, essential oils are recognized for their antimicrobial activities, which include antibacterial, antifungal, and antiviral effects. These properties are crucial in controlling pathogenic microorganisms and preserving food products. Essential oils, such as those derived from *A. graveolens* (dill), contain bioactive compounds like carvone, limonene, and dillapiole that exhibit potent antimicrobial effects. Carvone, for instance, has been shown to inhibit the growth of a broad spectrum of bacteria and fungi, making it effective in preventing food spoilage and extending shelf life (Kant and Kumar 2022). The antimicrobial activity of essential oils is often attributed to their ability to disrupt microbial cell membranes, interfere with enzyme systems, and inhibit metabolic processes. This bioactivity underscores the potential of essential oils as natural alternatives to synthetic preservatives in food safety and preservation (Ayub, Choobkar, et al. 2023).

*Anethum graveolens* , or dill, is a member of the Apiaceae family. This plant is commonly found in Central Asia, southern USSR, and the Mediterranean (Bailer et al. 2001). *A. graveolens* seeds are the oiliest part of the plant and are thus used to produce essential oil. The essential oil from dill has anti‐microbial, anti‐inflammatory, anti‐cancer, anti‐analgesic, gastro‐protective, anti‐diabetic, and anti‐fungal properties (Hamza 2017). The chemical composition of the essential oil obtained from dill depends on various factors, including its geographical source, the part of the plant used, the harvesting time, and the extraction method used. Different compounds obtained include flavonoids, coumarins, steroids, and phenolic acids (Khare 2004). In general, the chemical profile of *A. graveolens* contains carvone (C_10_H_14_), myristicin (C_11_H_12_O_3_), α‐phellandrene (C_10_H_16_), and d‐limonene (C_10_H_16_), and its leaves are high in steroids, terpenoids, and flavonoids (Ayub et al. 2018; Jana and Shekhawat 2010).

Essential oils, characterized by their special aroma, are complex, volatile substances produced as secondary metabolites by aromatic plants. Due to their antibacterial, virucidal, and anti‐fungal characteristics, as well as their fragrance, essential oils have been used in various applications in both the food and medicine industries. Essential oils are volatile, usually colored, and soluble in organic solvents and are less dense than water. They can be extracted from all parts of the plant, including leaves, fruit, seeds, roots, peels, and bark (Bakkali et al. 2008; de Sousa et al. 2023).

There are different extraction techniques employed for extracting essential oil from the different parts of the plant, some of which are regarded as conventional extraction methods, while more advanced extraction methods are now being adopted (Akhavan‐Mahdavi and Jafari 2024; Ayub, Choobkar, et al. 2023). Conventional methods of extraction include hydro distillation and steam distillation. In hydro distillation, the plant material is immersed in boiling water. The extracted oil is protected from overheating due to the presence of surrounding water (Jafari and Akhavan‐Mahdavi 2023). However, this technique has certain drawbacks, including higher energy costs, being time‐consuming, and potential chemical changes caused by the presence of water. On the other hand, steam distillation is more useful by comparison, resulting in higher yields of essential oils with a better chemical composition and purity (Sovová and Aleksovski 2006). However, in both cases, the yield of essential oil depends on several factors, including the amount of plant material used, the extraction time, the type of plant material used, and the volume of water used for the extraction. Additionally, the yield is influenced by the size and preparation of the raw plant material. It is crucial to optimize these parameters to achieve the maximum yield and desired quality of the essential oils (Boutekedjiret et al. 2003).

CO_2_ extraction, supercritical CO_2_ extraction, and SCWE are considered more advanced methods of extraction. SCF‐CO_2_ extraction is an emerging technique that provides superior extraction with lower use of organic solvents and requires moderate temperatures. This results in higher purity and eliminates the need for additional cleanup steps. CO_2_ is commonly used as a supercritical fluid for extracting essential oils because it is non‐explosive and volatile, making it easy to eliminate. Additionally, CO_2_ is low in viscosity, has a low latent heat of evaporation, is non‐toxic, and is readily available. By employing the most advanced technique, response surface methodology (RSM), various parameters such as pressure, temperature, flow rate, and extraction time can be optimized to enhance the efficiency of the extraction process (Mirbagheri et al. 2023).

SCWE used to extract essential oils, employing high‐temperature water as a solvent. Temperatures for SCWE range from 100°C to 374°C, with high pressure applied to maintain water in a liquid state. SCWE enhances mass transfer through both convection and diffusion processes. Additionally, adjusting the temperature and pressure during this method allows for the control of the dielectric constant and polarity of the sample (Zhang et al. 2020). SCWE is preferred over other techniques due to its low cost, simplicity, reduced chances of chemical degradation, better environmental compatibility, and shorter extraction time (Ozel et al. 2003).

Superheated steam extraction (SHSE) is another emerging technique used for extracting essential oils from various plant parts. In SHSE, the temperature of the steam at a specific pressure is higher than the saturation temperature (Rouatbi et al. 2007). Superheated steam is produced by increasing the temperature of normal steam (Ayub, Choobkar, et al. 2023). This method carries a significant amount of thermodynamic energy as latent heat of vaporization. Due to its high thermal conductivity, low oxygen conditions, enhanced extraction capacity, and prevention of oxidation in extracted components, SCWE is preferred over conventional techniques. The increased yield of essential oils extracted through SHSE might be due to a decrease in polarity and dielectric constant because of the higher steam temperature (Ayub, Goksen, et al. 2023). This technique was used in the drying and baking industry, but it is now applied to oil extraction. SHSE has been used for extracting essential oils from thyme ( *Thymus vulgaris* ), black pepper ( *Piper nigrum* ), *Boswellia serrata* *oleo* gum resins, *Pinus roxburghi* oleoresin, and *Syzygium aromaticum* (Rouatbi et al. 2007). Previous studies indicate that hydro distillation and steam distillation have been used to investigate the chemical composition and biological activities of essential oils from *A. graveolens* seeds (Garcez et al. 2020; Kokotkiewicz et al. 2021; Mohammed et al. 2019; Nouioura et al. 2024). However, the use of SHSE has not yet been explored for extracting essential oils from these seeds and its effect on the biological activities of the resulting essential oil. This study examined the variations in the chemical composition and biological activities of essential oil extracted from *A. graveolens* seeds using various extraction protocols: hydro distillation, steam distillation with advanced SFE, and SHSE.

## 2 Material and Methods

### 2.1 Collection of Plant Material and Extraction

*Anethum graveolens* seeds were collected from a botanical garden in Pakistan. Dr. Fahim Arshad from the University of Okara, Pakistan, confirmed the identity of the plant material. Seeds were dried in the shade, and the EO was extracted by using four techniques, i.e., HD, SD, SC‐CO_2_, and SHS extraction.

### 2.2 Extraction Methods

#### 2.2.1 Hydro Distillation

Hydro distillation was performed using a Clevenger apparatus, which included a heating mantle, a 5000 mL round‐bottom flask, a condenser, a Dean‐Stark trap, and a separating funnel. Three hundred grams of A. graveolens seeds were submerged in 3000 mL of distilled water in the flask. The mixture was heated to boiling, and the resulting vapor was condensed using a condenser. The essential oil was separated from the aqueous layer using a separating funnel. Anhydrous sodium sulfate (5 g) was added to the oil to absorb moisture, and the sample was filtered through a 0.22‐µm microfilter (Sigma‐Aldrich, Germany). The extraction process was conducted for three hours and repeated three times for consistency (Ayub, Goksen, et al. 2023).

#### 2.2.2 Steam Distillation

The apparatus for SD comprised a round‐bottom flask, biomass vessel, heating mantle, condenser, thermometer, and separating funnel. In SD, ground 100 g of *A. graveolens* seeds were added to the flask and 3000 mL of water was added. This assembly was placed on the heating mantle and brought to a boil. Steam generated by the boiling water interacted with the plant material, resulting in the essential oil extraction. Upon condensing the oil and water vapors, the extract was collected in a separating funnel. After separating the oil from the aqueous layer, anhydrous sodium sulfate (5 g) was added to the oil to absorb any moisture and was passed through the microfilters (Sigma‐Aldrich, Germany) (Azeem et al. 2022).

#### 2.2.3 Supercritical Carbon Dioxide Extraction

The extraction system for this method consisted of a high‐pressure pump and a supply of liquid CO_2_. Before the extraction, the liquid CO_2_ was chilled to 10°C and introduced into an extraction tank pre‐set to a temperature of 45°C. Throughout the process, pressure control was maintained with an accuracy of ±1%, ensuring precise operating conditions. Approximately 500 g of plant seeds were placed in the extraction vessel, mixed with an equal weight of cotton wool to optimize the diffusion of CO_2_. This arrangement allowed supercritical CO_2_ to permeate and dissolve into the plant matrix. The movement of supercritical CO_2_ through the plant material was regulated by a heated micrometer valve, which managed the release of CO_2_ containing the dissolved plant compounds into the collection system. Upon exiting the valve, the SCF–CO_2_ was decompressed to ambient pressure, allowing the extracted compounds to precipitate in a collection vessel maintained at room temperature (25°C) and pressure (1 bar). Anhydrous sodium sulfate was added to the collected extracts to remove any residual moisture. The volume of CO_2_ used in each extraction was quantified with a calibrated wet test meter (W‐NK, Ritter Apparatebau GmbH & Co. KG, Germany) under 25°C and 1 bar (ambient pressure). The CO_2_ flow rate was set at 2 L/min. The weight of the extracted oils was accurately determined using an analytical balance. This methodological setup provided a controlled environment for the effective extraction of essential oils using supercritical CO_2_, ensuring reproducibility and efficiency in the extraction process (Rajput et al. 2023).

#### 2.2.4 Superheated Steam Distillation

Superheated steam distillation (SHSD) equipment (Model: SHSD‐001, PAMICO Technologies, Faisalabad, Punjab, Pakistan) was also used to extract the essential oil from dill seeds. The equipment comprised a superheated steam (SS) generator, an SS biomass extraction chamber, SS condensers and chiller, and a glass hydrosol collection vessel. Plant seeds (3000 g) were placed in the extraction chamber, and the temperature of the superheated steam was closely monitored using thermocouples attached to the steam supply line. The steam was generated at a pressure of 5.17 bar and a temperature of 150°C. After 60 min of exposure to the superheated steam, 0.25–0.50 g of anhydrous Na_2_SO_4_ was added to the extracted essential oil to remove any moisture. The oil was then filtered using a 0.22‐μm microfilter (Millex‐GV, Sigma‐Aldrich, Germany) and stored in amber glass vials (VWR International, USA) for further analysis.

To correct for the different amounts of starting material used between extraction methods, the yields of essential oil were standardized based on the weight of the plant material. The yield from each method was calculated as a percentage of the initial plant material weight, allowing for a direct comparison of the efficiency of each extraction technique regardless of the starting material quantity. To ensure the reliability of the findings, the extraction process was repeated three times.

### 2.3 Antimicrobial Activity

#### 2.3.1 Preparation of Microbial Cultures for Antimicrobial Assays

To assess the antimicrobial activity of *A. graveolens* essential oil (EO) extracted by different methods, microbial strains were obtained from a recognized culture collection, specifically *E. coli* (ATCC 25922), *S. aureus* (ATCC 25923), *Candida albicans* (ATCC 10231), and *Aspergillus niger* (ATCC 16404). These strains were selected due to their relevance as common pathogens and their frequent use in antimicrobial studies.

#### 2.3.2 Culture Media and Growth Conditions

To prepare the microbial cultures for the antimicrobial assays, strains were initially obtained from a reputable culture collection and grown in specific media under optimal conditions, such as nutrient broth for bacteria like *E. coli* and *S. aureus* , and yeast extract‐peptone‐dextrose (YPD) broth for fungi like *C. albicans* . Cultures were incubated at appropriate temperatures (typically 37°C for bacteria and 25°C or 30°C for fungi) until reaching the log phase. They were then standardized to a concentration of approximately 10^6^ colony‐forming units (CFU)/mL. For the assay, molten nutrient agar or suitable media, cooled to around 45°C, was mixed with the standardized microbial cultures immediately before pouring. This mixture was poured into petri dishes and allowed to solidify at room temperature. Control plates without antimicrobial agents were prepared to verify microbial growth and the effectiveness of the medium. The entire procedure was conducted in triplicate to ensure reproducibility and accuracy of the results.

The method was used to determine the MIC values of essential oils specifically for various bacterial strains. This involved preparing serial dilutions of the essential oils in suitable growth media and inoculating them with bacterial cultures. The MIC was determined as the lowest concentration of the essential oil that inhibited visible growth of the bacteria after incubation.

To prepare fungal suspensions for an antifungal assay, first grow the fungal strains on a suitable agar medium (e.g., Sabouraud Dextrose Agar) at the appropriate temperature until they reach the desired growth stage. Next, harvest the fungal spores by transferring colonies to a sterile container with sterile physiological saline, then vortex or shake the container to create a uniform spore suspension. Adjust the concentration of the suspension by measuring its optical density (OD) with a spectrophotometer, aiming for an OD of around 0.1 to 0.2, or by counting spores directly using a hemocytometer if a precise concentration is needed. Dilute or concentrate the suspension as necessary to achieve the target spore concentration. For the assay, inoculate the surface of agar plates with the fungal suspension using a sterile spreader or add it to wells in a microtiter plate for broth microdilution. Include positive and negative controls to validate results. After incubation under appropriate conditions (typically 25°C–30°C for 24–48 h), measure the zone of inhibition for disk diffusion assays or assess growth in broth dilution assays to determine the minimum inhibitory concentration (MIC).

#### 2.3.3 Agar Well Diffusion Method

The agar well diffusion method was used to evaluate the antimicrobial activity of the extracted essential oils (23). The microbial strains used in this study included *E. coli* (ATCC 25922), *S. aureus* (ATCC 25923), *Bacillus subtilis* (ATCC 6633), *Pasteurella multocida* (ATCC 43137), *Fusarium solani* (ATCC 36031), *A. niger* (ATCC 16404), *Alternaria alternata* (ATCC 16862), and *Aspergillus flavus* (ATCC 9643). These strains were sourced from the American Type Culture Collection (ATCC).

Microbial strains were added to 25 mL of growth medium solution—Mueller–Hinton agar for bacteria and Sabouraud dextrose agar for fungi—and incubated overnight. The concentration of the suspension was then adjusted to 10^8^ colony‐forming units (CFU) per mL. The contents were mixed with the culture medium and poured into Petri dishes. After the medium solidified, wells were cut into the agar using a sterilized cork borer, and 10 μL of the essential oil solution (10 mg of essential oil was dissolved in 1 mL of 10% dimethyl sulfoxide) extracted by various methods, including SHSE, was added to the wells.

A standard antimicrobial agent, ciprofloxacin for bacteria and fluconazole for fungi, was included as a control to test antibacterial and antifungal activity. The Petri dishes were incubated at specific temperatures to assess microbial activity (for antibacterial activity incubated for 24 h at 37°C and for antifungal activity: incubated for 40 h at 30°C). A digital Vernier caliper was used to measure the zones of inhibition that resulted following the incubation (18). This procedure was repeated three times to ensure the reproducibility of the results.

#### 2.3.4 Resazurin Microtitre Plate Assay

The resazurin microtitre plate assay, as described by Sarker et al. (2007), was used to determine the MIC value of essential oils for the various microbial strains. To prepare the sample, 10 mg of essential oil was dissolved in 1 mL of 10% dimethyl sulfoxide (DMSO) (Sigma‐Aldrich, USA). The resazurin indicator solution was prepared by dissolving 27 mg of resazurin in 4 mL of deionized water.

In the first row of 96‐well plates, 100 μL of the sample solution and the antibiotic ampicillin (at a concentration of 100 μg/mL) were pipetted. Fifty microliters of nutrient broth were added to all wells except those in the first row. A two‐fold serial dilution was performed so that each well contained 50 μL of the sample. Then, 10 μL of the resazurin solution and 30 μL of iso‐sensitized broth at a strength of 3.3× were added to all wells. The plates were incubated for 24 h at 37°C. Following the incubation, the MIC values were determined visually. A color change from purple to pink or colorless indicated bacterial growth. The MIC value was identified at the concentration where the color change was first observed.

#### 2.3.5 Micro‐Dilution Broth Susceptibility Assay

The micro‐dilution broth susceptibility analysis was used to determine the MIC of EO extracted by various methods against fungal strains, as described by (Dabur et al. 2004). Ten milligrams of EO were dissolved in 1 mL of 10% DMSO (Sigma‐Aldrich, USA). For the standard, 1 mL of fluconazole was added to 1 mL of a 10% DMSO solution.

In the first row of a 96‐well plate, 100 μL of both the standard and sample solutions was added. All other wells received 50 μL of dextrose broth, except for the first row. Subsequently, a two‐fold serial dilution was performed so that each well contained 50 μL of the sample. Then, 20 μL of the microorganism suspension and 130 μL of Sabouraud solution were added to all wells. The plates were incubated at 30°C for 48 h. The MIC value was determined visually at the concentration where complete inhibition of fungal growth was observed.

### 2.4 GC–MS Analysis

GC–MS analysis was employed to determine the volatile components extracted through various methods. A DB‐5 capillary column (50 m × 0.25 mm, thickness 0.25 μm) and a mass detector were used in the Shimadzu gas chromatograph GC‐2010 system. Essential oils were diluted with n‐hexane at a ratio of 1:10 and utilized as the injection volume. The sample was injected into the injection port using an injection syringe. The GC column was initially heated to 60°C for 3 min. The temperature of the column was then gradually increased to 240°C at a constant heating rate of 24°C per minute for 10 min. Nitrogen gas, at a flow rate of 1.5 mL per minute, served as the carrier gas. The temperature of the mass spectrometer transfer line was maintained at 240°C. The mass spectrometer detector operated in electron ionization mode at 70 eV. Retention indices were determined by running standards of n‐alkanes. The retention indices and mass spectra results were compared with data from the Pherobase mass spectral database and the NIST library (Ayub et al. 2018). Co‐injection with authentic standards was performed to confirm the compounds. A quantitative analysis of EO components was conducted (Das et al. 2019). RF values were calculated using the following equation:

$${\mathrm{RF}}_{c}=\left(A_{c}/A_{\text{is}}\right)/\left(C_{c}/C_{\text{is}}\right)$$

RF represents the response factor of components of EO. *A*_c_ and *A*_is_ represent the peak area of components of EO and the internal standard, respectively. Concentrations of components of the essential oil and internal standard are represented by *C*_c_ and *C*_is_, respectively. The percentage of each component of the essential oil was calculated by the RFs.

Corrected area peak area for the component/response factor for that component.

$$\begin{array}{c} \text{Percentage}=(\text{corrected area for the component}/\\ \;\text{total of the corrected areas})\times 100 \end{array}$$

### 2.5 Antioxidant Assays

#### 2.5.1 DPPH Free Radical Scavenging Activity

The antioxidant activity of the essential oils was evaluated using the DPPH (2,2‐diphenyl‐1‐picrylhydrazyl) radical scavenging assay. For this assay, a standard solution of Butylated Hydroxytoluene (BHT) was prepared as the reference antioxidant. BHT was dissolved in ethanol to create a standard solution at a concentration of 100 μg/mL. This solution was used to compare the antioxidant activity of the essential oils. The essential oil samples and BHT solution were added to a DPPH solution, and the reduction in DPPH absorbance was measured at 517 nm after 30 min of incubation. The percentage inhibition of the DPPH radical was calculated to determine the antioxidant activity of the essential oils relative to the BHT standard (Mensor et al. 2001). DPPH scavenging activity was calculated by using the following formula:

$$\text{DPPH scavenging activity}\%=100-\left[\left\langle \frac{{\mathrm{Abs}}_{\text{sample}}}{\;{\mathrm{Abs}}_{\text{control}}\;}\right\rangle \times 100\right]$$

#### 2.5.2 Reducing Power Ability (RPA) of the Essential Oil

The antioxidant activity of essential oils was assessed using the DPPH (2,2‐diphenyl‐1‐picrylhydrazyl) radical scavenging assay. A standard solution of Butylated Hydroxytoluene (BHT) was prepared in ethanol at a concentration of 100 μg/mL, and this standard was used to benchmark the antioxidant activity. Essential oil samples were similarly prepared by dissolving them in ethanol to achieve a concentration of 100 μg/mL. For the assay, 1 mL of each essential oil sample solution or the BHT standard solution was mixed with 1 mL of a 0.1 mM DPPH solution in ethanol, which was freshly prepared and kept in the dark. The mixture was incubated at room temperature in the dark for 30 min to allow the reaction to occur. After incubation, the absorbance was measured at 517 nm using a UV–Vis spectrophotometer. The percentage inhibition of the DPPH radical was calculated using the formula (Yasin et al. 2021):

$$\text{Percentage Inhibition}=\frac{\left(\;A_{\text{control}}-A_{\text{sample}}\right)}{\;A_{\text{control}}}\times 100$$

where *A*_control_ is the absorbance of the DPPH solution without any antioxidant, and *A*_sample_ is the absorbance of the DPPH solution with the essential oil or BHT. This procedure allows for the comparison of antioxidant activity between the essential oils and the BHT standard.

In the DPPH radical scavenging assay, ethanol was used as the control, mixed with DPPH solution to measure the baseline absorbance without any antioxidant. Butylated Hydroxytoluene (BHT) served as the standard antioxidant, with a standard solution prepared in ethanol at a concentration of 100 μg/mL.

#### 2.5.3 Hydrogen Peroxide Scavenging Activity

The H_2_O_2_ scavenging activity of essential oils was assessed using a spectrophotometer. A 2 mM hydrogen peroxide solution was prepared with a 0.17 M phosphate buffer at pH 7.4. Various concentrations of the essential oil solution (600 mL) and 0–10 mg/mL of ascorbic acid were added to the H_2_O_2_ solution. After incubating the samples for 10 min, the absorbance was measured at 230 nm (Senthilkumar et al. 2020).

$$\begin{array}{c} \%\mathrm{Age}\;\text{of hydrogen peroxide scavenged}=\\ \left[\mathrm{Abs}\;\text{of control}-\mathrm{Abs}\;\text{of sample}\right]\times 100 \end{array}$$

### 2.6 Statistical Analysis

STATISTICA 5.5 (StatSoft Inc., Tulsa, OK, USA) was used for the ANOVA statistical analysis of data, and all the experiments were performed three times. A *p*‐value of less than 0.05 indicates statistical significance. Results are shown as mean values along with standard deviation based on triplicate observations.

## 3 Results and Discussion

### 3.1 Essential Oil Yield

**FIGURE 1:**
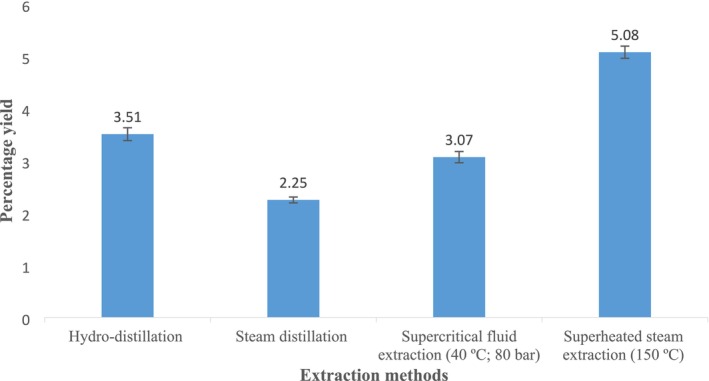
Percentage yield of *Anethum graveolens* essential oil extracted using different extraction methods.

The yields of essential oil extracted from the *A. graveolens* seeds using HD, SD, SCF‐CO_2_, or superheated steam extraction oil are given in Figure 1. The study reveals that the highest essential oil yield, up to 5.08% ± 0.13%, was achieved through superheated steam extraction, while the lowest yield, approximately 2.25% ± 0.05%, was obtained via steam extraction. These results align with existing literature on steam distillation of *A. graveolens* seeds, which produces a lesser yield of essential oil than hydro distillation, with recorded yields up to 0.66% (Badar et al. 2008). In Thailand, yields from dill seeds were 1.05% and 2.01% for hydro and steam distillation, respectively (Ruangamnart et al. 2015). Similarly, dill seeds from China provided a 3.8% yield using hydro distillation (Yili et al. 2006). Further review of previous studies indicates that variations in harvest timing and seeding dates impact the yield of essential oils extracted via hydro distillation, with yields ranging from 0.2% to 4.6% (Bowes et al. 2004). Additionally, the duration of the extraction process also influences the yield of essential oils. The age of the seedling post germination also plays a significant role in the yield of essential oils.

Essential oil obtained through superheated steam extraction produced the highest yield, reaching 5.08% ± 0.13%. Previous studies, such as those by Li et al. (2019), show that supercritical CO_2_ extraction under optimal temperature, pressure, and particle size can achieve even higher yields, up to 6.70% (Li et al. 2019). The effectiveness of superheated steam extraction may be attributed to its enhanced penetration capabilities and stable energy output (Ayub, Goksen, et al. 2023). Additionally, increasing the extraction temperature in subcritical water extraction methods has been found to improve yields in *Citrus hysteria* leaves, primarily due to changes in the water's physical properties such as reduced density, viscosity, hydrogen bonding, polarity, and surface tension (Halim et al. 2021).

The extraction conditions used in this study, including temperature and pressure, were selected based on established methods for essential oil extraction. According to the literature, higher yields of essential oils are often achieved with optimized conditions such as higher temperatures or pressures in methods like supercritical CO_2_ extraction (SFE) (Mirbagheri et al. 2023). For example, studies have shown that increasing the extraction temperature and optimizing pressure settings can enhance oil yields (Sovová and Aleksovski 2006; Zhang et al. 2020). In our study, we aligned our extraction parameters with these recommendations to ensure high yields and efficiency.

The age of the seedlings post‐germination plays a crucial role in the yield of essential oils, as the concentration and composition of essential oils can vary with plant maturity. For instance, older seedlings might have higher or more varied essential oil content compared to younger ones (Bailer et al. 2001). In our study, we focused on mature seeds for extraction to standardize the essential oil yield, as younger seedlings were not included to avoid variability in the results.

These findings suggest that optimizing temperature and pressure settings can significantly enhance essential oil yields. For instance, dill seeds yield higher amounts of essential oil (up to 5.08% ± 0.13%) compared to thyme and black pepper, which yield 1.70% and 2.80%, respectively (Rouatbi et al. 2007). Although superheated steam extraction is not widely used for extracting essential oils, it has been proven to yield a higher percentage of oil due to the elevated temperatures involved. This method results in a significantly higher yield for *A. graveolens* compared to essential oils extracted from *Boswellia* species such as *B. elongata, B. dioscorides, B. carteri*, and *B. socotrana*, using the hydro‐distillation method at 100°C and normal pressure, which typically yields between 0.2% and 0.5% (33). Furthermore, the yield from *Cinnamomum zeylanicum* extracted using superheated water was notably lower than that of the current study (Jayawardena and Smith 2010). Overall, a comparison of extraction techniques indicates that superheated steam extraction, followed by hydro distillation, supercritical CO_2_ extraction, and steam distillation, produces the highest yields. Thus, superheated steam extraction is a superior method for obtaining higher yields of essential oils.

### 3.2 Antimicrobial Activity

The well diffusion assay, resazurin microtiter plate assay, and microdilution broth assay were utilized to evaluate the antimicrobial properties of essential oils from *A. graveolens* seeds after extraction. Tables 1 and 2 outline the antimicrobial activity of *A. graveolens* essential oil, showing inhibition zones ranging from 10.21 to 28.06 mm and MIC values between 0.04 and 5.00 mg/L. The positive control's MIC and inhibition zones range from 0.02 to 0.63 mg/L and 31.06 to 39.21 mm, respectively. These results show that the essential oil extracted via superheated steam extraction exhibits the highest antimicrobial activity, demonstrated by the widest inhibition zones (14.16–28.06 mm) and the lowest MIC values (0.04–0.65 mg/L). Variations in the antimicrobial effectiveness of *A. graveolens* essential oil may stem from the different extraction methods used (Glišić et al. 2007).

**TABLE 1:** Inhibition zone values (mm) of *Anethum graveolens* essential oils against bacterial and fungal strains.

Microorganism	Essential oil isolated through different extraction method				Positive control^a^
	Hydro‐distillation	Steam distillation	Supercritical fluid extraction	Superheated steam distillation	
*Staphylococcus aureus*	13.04 ± 0.06^e^	16.50 ± 0.09^d^	17.58 ± 0.11^c^	18.03 ± 0.12^b^	31.06 ± 0.15^a^
*Escherichia coli*	15.01 ± 0.09^e^	16.10 ± 0.11^d^	18.34 ± 0.10^c^	19.08 ± 0.12^b^	38.01 ± 0.31^a^
*Bacillus subtilis*	15.18 ± 0.04^e^	18.65 ± 0.07^d^	20.36 ± 0.08^c^	21.12 ± 0.09^b^	35.12 ± 0.17^a^
*Pasteurella multocida*	23.10 ± 0.12^e^	25.04 ± 0.14^d^	26.72 ± 0.13^c^	28.06 ± 0.10^b^	39.21 ± 0.28^a^
*Fusarium solani*	11.72 ± 0.06^e^	12.92 ± 0.14^d^	13.78 ± 0.12^c^	15.66 ± 0.16^b^	34.43 ± 0.12^a^
*Aspergillus niger*	10.21 ± 0.09^e^	11.40 ± 0.11^d^	13.45 ± 0.10^c^	14.16 ± 0.12^b^	33.04 ± 0.31^a^
*Alternaria alternate*	15.21 ± 0.04^e^	16.62 ± 0.08^d^	17.98 ± 0.09^c^	19.04 ± 0.06^b^	38.02 ± 0.12^a^
*Aspergillus flavus*	11.32 ± 0.12^e^	12.24 ± 0.15^d^	13.74 ± 0.13^c^	15.28 ± 0.05^b^	34.11 ± 0.11^a^

*Note:* Values are mean ± standard deviations of three separate determinations. Different superscript letters represent significant differences among *Anethum graveolens* essential oils extracted by different extraction methods.

^a^ Positive control for bacteria and fungi was Amoxicillin and Fluconazole (25 μg/disc), respectively.

**TABLE 2:** Minimum inhibitory concentration (MIC) of *Anethum graveolens* essential oils against bacterial and fungal strains.

Microorganism	Essential oil isolated through different extraction method				Positive control^a^
	Hydro‐distillation	Steam distillation	Supercritical fluid extraction	Superheated steam distillation	
*Staphylococcus aureus*	5.00 ± 0.05^a^	2.50 ± 0.05^b^	1.25 ± 0.03^c^	0.63 ± 0.01^d^	0.078 ± 0.00^e^
*Escherichia coli*	5.00 ± 0.05^a^	2.50 ± 0.05^b^	1.25 ± 0.03^c^	0.63 ± 0.02^d^	0.31 ± 0.01^e^
*Bacillus subtilis*	2.50 ± 0.03^a^	0.63 ± 0.03^b^	0.63 ± 0.02^b^	0.31 ± 0.00^c^	0.039 ± 0.01^d^
*Pasteurella multocida*	0.63 ± 0.03^a^	0.31 ± 0.03^b^	0.16 ± 0.02^c^	0.08 ± 0.03^d^	0.02 ± 0.00^e^
*Fusarium solani*	2.50 ± 0.01^a^	1.25 ± 0.01^b^	0.63 ± 0.02c	0.31 ± 0.03^d^	0.16 ± 0.01^e^
*Aspergillus niger*	5.00 ± 0.05^a^	5.00 ± 0.05^a^	2.50 ± 0.03^b^	1.25 ± 0.02^c^	0.63 ± 0.01^d^
*Alternaria alternate*	0.63 ± 0.02^a^	0.63 ± 0.00^a^	0.16 ± 0.04^b^	0.04 ± 0.03^c^	0.02 ± 0.00^d^
*Aspergillus flavus*	2.50 ± 0.00^a^	2.50 ± 0.00^a^	1.25 ± 0.02^b^	0.65 ± 0.03^c^	0.32 ± 0.00^d^

*Note:* Values are mean ± standard deviations of three separate determinations. Different superscript letters represent significant differences among *Anethum graveolens* essential oils extracted by different extraction methods.

^a^ Positive control for bacteria and fungi was Amoxicillin and Fluconazole (25 μg/disc), respectively.

This essential oil was tested against both gram‐positive and gram‐negative bacteria, including *S. aureus, B. subtilis, E. coli*, and *P. multocida* . The MIC values for these bacteria ranged from 0.08 to 5.00 mg/L. *P. m* *ultocida*, a Gram‐negative strain, showed the highest susceptibility to the antimicrobial agent with an inhibition zone of 28.08 mm, while *E. coli* had an inhibition zone of 19.08 mm. Among Gram‐positive bacteria, *B. subtilis* exhibited greater susceptibility with an inhibition zone of 21.12 mm compared to *S. aureus* at 18.03 mm. These results are in good agreement with a previous study, which found that *B. serrata* oleo‐gum‐resin essential oil exhibited higher susceptibility against Gram‐negative strains *P. multocida* and *E. coli* compared to Gram‐positive strains *B. subtilis* and *S. aureus* (Ayub et al. 2018 #393). Essential oils exhibit antimicrobial effects by increasing cell membrane permeability, leading to leakage. This enhanced permeability is associated with ion loss, a decrease in membrane potential, disruption of the proton pump, and ultimately, the collapse of the ATP pool. These disruptions to the cell membrane can trigger further damage to other cellular structures. Essential oils penetrate these compromised membranes and modify the structural composition of phospholipids, fatty acids, and polysaccharides. Additionally, they may attach to cytoplasmic components, disrupting protein and lipid layers. The hydrophobic elements within the essential oils interfere with cellular functions by interacting with lipids in the mitochondria and the cell membrane, contributing to their antimicrobial activity (Akthar et al. 2014).

These findings corroborate previous research indicating the antibacterial properties of *A. graveolens* essential oil. Notably, studies have demonstrated its effectiveness against *S. aureus* and *B. subtilis* in Bulgarian samples (Jirovetz et al. 2003) and against *Salmonella typhi, B. subtilis, S. aureus*, and *E. coli* using steam distillation (Badar et al. 2008). Furthermore, *S. aromaticum* essential oil, extracted via superheated steam, also showed significant antibacterial activity against *P. multocida* , with an inhibition zone of 26.87 mm (Ayub, Hanif, et al. 2023). Earlier research highlighted the antimicrobial potential of *A. graveolens* essential oil against *S. aureus*, attributed to its content of monoterpenes and sesquiterpenes (Yili et al. 2009).

The antifungal properties of *A. graveolens* essential oil extracts are documented in Tables 1 and 2. The efficacy of dill seed essential oil was evaluated against several fungal strains, including *F. solani*, *A. alternata* , *A. niger*, and *Aspergillus flavus*. The inhibition zones and MIC values ranged between 10.21–19.04 mm and 0.04–5.00 mg/L, respectively. *Alternaria alternata* showed the highest inhibition, with a zone of 19.04 mm and an MIC of 0.04 mg/L. Conversely, the lowest antifungal potential was observed in *A. niger* , which displayed an inhibition zone of 10.21 mm and an MIC value of 5.00 mg/L.

This data aligns with prior findings that *A. graveolens* has significant antimicrobial capabilities, attributed to the presence of carvone and limonene as its main components (Chahal et al. 2017). These substances are noted for their antibacterial effectiveness against both gram‐positive and gram‐negative bacterial strains (Purkayastha et al. 2012). Furthermore, it was determined that the antibacterial potency of dill seed oil surpasses that of dill leaf oil (Rasheed et al. 2010). Studies have shown that carvone in dill seed oil exhibits the most potent antibacterial and antifungal activities, with MIC values ranging from 0.5–4.0 μg/mL, while limonene demonstrated antimicrobial activity with MIC values between 1.0–9.0 μg/mL (Kazemi et al. 2012). Antifungal assessments on various strains including *A. flavus, A. niger, A. ochraceus, F. oxysporum f*. sp. *Albedinis*, *Penicillium expansum*, *A. alternata* , and *Cladosporium* species revealed that dill seed EO exhibited broad‐spectrum antifungal effects against these pathogens (Khaldi et al. 2015). This corroborates the reported mechanisms, highlighting the role of limonene and d‐carvone in inhibiting fungal sporulation and spore germination (Chahal et al. 2017). A close review of the literature confirmed our findings that the major compounds d‐limonene, carvone, and dillapiole found in the essential oil extracted from *A. graveolens* are indeed responsible for its antioxidant and antimicrobial activity. However, Minor compounds, myristicin [1] and thymol [2], might also contribute to the little antimicrobial effects as well. Similarly, the minor compound α‐terpineol might be responsible for the antioxidant activity of the *A. graveolens* essential oil. However, a thorough review of the literature shows that no synergistic effects between the essential oil components have been reported so far.

### 3.3 Chemical Composition of Essential Oil

The chemical composition of dill seeds essential oil extracted by the various extraction methods was characterized by GCMS, the results of which are given in Table 3. The primary constituents of *A. graveolens* essential oil included 4‐Carene (1.23%–1.59%), d‐limonene (19.48%–23.01%), α‐thujene (3.12%–3.8%), trans dihydrocarvone (1.55%–4.87%), dillapiole (13.87%–17.04%), d‐carvone (38.01%–42.03%), and phytol (0.45%–3.37%). GCMS analysis revealed that EO extracted via HD and SD exhibits lower concentrations of d‐limonene and d‐carvone compared to those extracted by SCF‐CO_2_ and SHSE, where d‐limonene levels were found at 22.61% and 23.01%, and d‐carvone at 41.56% and 42.03%, respectively. Additionally, dillapiole, another major component, showed higher concentrations in SHSE, while the other compounds were present in lower concentrations.

**TABLE 3:** Chemical composition of *Anethum graveolens* essential oils determined by GC–MS.

Components	RI^A^	RI^B^	% Composition of essential oil				Identification method
			Hydro‐distillation	Steam distillation	Supercritical CO_2_ extraction	Superheated steam distillation	
α‐Thujene	931	927	3.71 ± 0.02^b^	—	3.12 ± 0.00^c^	3.80 ± 0.03^a^	a, b
Sabinene	976	973	0.18 ± 0.01^b^	0.05 ± 0.00^d^	0.12 ± 0.01^c^	0.23 ± 0.03^a^	a, b
β‐pinene	980	977	0.75 ± 0.00^c^	0.80 ± 0.00^b^	0.68 ± 0.01^d^	0.85 ± 0.03^a^	a, b
β‐myrcene	984	983	0.23 ± 0.01^b^	0.19 ± 0.01^b^	0.20 ± 0.02^b^	0.25 ± 0.00^a^	a, b
4‐Carene	1001	1007	1.23 ± 0.01^c^	1.46 ± 0.03^b^	1.58 ± 0.02^a^	1.59 ± 0.03^a^	a, b
d‐limonene	1031	1029	19.48 ± 0.02^d^	21.96 ± 0.02^c^	22.61 ± 0.03^b^	23.01 ± 0.04^a^	a, b
m‐cymene	1029	1022	1.98 ± 0.01^c^	2.09 ± 0.03^b^	2.28 ± 0.03^a^	2.30 ± 0.04^a^	a, b
1,5,8‐p‐menthatriene	1119	1112	0.26 ± 0.00^c^	0.36 ± 0.00^b^	0.42 ± 0.00^a^	0.45 ± 0.03^a^	a, b
α‐terpineol	1186	1184	—	0.57 ± 0.00^b^	—	0.60 ± 0.01^a^	a, b
*trans*‐dihydrocarvone	1193	1192	3.49 ± 0.03^c^	4.87 ± 0.04^a^	2.66 ± 0.01^b^	1.55 ± 0.02^d^	a, b
*cis*‐dihydrocarvone	1194	1201	0.96 ± 0.01^c^	2.72 ± 0.03^a^	1.93 ± 0.02^b^	0.45 ± 0.02^d^	a, b
d‐carvone	1254	1242	38.01 ± 0.03^d^	40.73 ± 0.02^c^	41.56 ± 0.05^b^	42.03 ± 0.04^a^	a, b
Thymol	1290	1290	0.12 ± 0.00^c^	0.18 ± 0.00^b^	0.27 ± 0.01^a^	0.30 ± 0.04^a^	a, b
Geranyl acetone	1389	1380	0.53 ± 0.00^d^	0.84 ± 0.02^b^	0.66 ± 0.00^c^	0.90 ± 0.01^a^	a, b
β‐Caryophyllene	1417	1419	0.56 ± 0.02^a^	0.49 ± 0.04^b^	0.50 ± 0.01^b^	0.60 ± 0.03^a^	a, b
Caryophyllene	1428	1420	0.13 ± 0.01^c^	0.17 ± 0.01^b^	0.15 ± 0.01^b^	0.19 ± 0.00^a^	a, b
*Trans*‐α‐bergamotene	1432	1434	0.90 ± 0.12^a^	0.01 ± 0.00^c^	—	0.03 ± 0.00^b^	a, b
Bisabolene	1491	1499	0.24 ± 0.01^b^	0.19 ± 0.01^c^	0.21 ± 0.01^c^	0.28 ± 0.00^a^	a, b
Anethole	1283	1285	2.09 ± 0.02^c^	2.28 ± 0.01^b^	2.58 ± 0.02^a^	2.30 ± 0.01^b^	a, b
Myristicin	1520	1518	2.51 ± 0.04^a^	1.20 ± 0.03^c^	1.12 ± 0.03^d^	1.53 ± 0.02^b^	a, b
Dillapiole	1619	1621	13.87 ± 0.01^d^	15.98 ± 0.03^c^	16.56 ± 0.01^b^	17.04 ± 0.00^a^	a, b
Phytol	2086	2099	1.87 ± 0.01^c^	2.86 ± 0.03^b^	3.37 ± 0.04^a^	0.45 ± 0.02^d^	a, b

*Note:* Mean ± SD. Various letters in superscripts show significant differences among *Anethum graveolens* essential oils extracted through different extraction techniques.

Abbreviations: a, identification based on retention index; b, identification based on comparison in mass spectra; RI^A^, retention indices observed against n‐alkanes; RI^B^, retention indices as reported in literature.

It has been noted that SHSE has not yet been applied for extracting essential oil from *A. graveolens* seeds. Consequently, the chemical composition results are compared with oils extracted using other methods. These results align closely with previously published data, indicating that dill seed essential oil typically has high concentrations of d‐limonene, d‐carvone, and dillapiole. However, essential oils obtained through steam distillation are found to have higher levels of carvone and limonene (Chahal et al. 2017). The antimicrobial effectiveness of *A. graveolens* seed EO has been studied, with findings suggesting that carvone and limonene contribute to its antifungal properties (Ma et al. 2015). Another study showed that EO from dill seeds grown in Uzbekistan contains up to 73% carvone and 14.69% limonene, corroborating previous findings (Yili et al. 2009). Egyptian dill seed essential oil, comprising 21 components including dillapiole, dihydrocarvone, α‐phellandrene, limonene, and carvotanacetone, was found to have a composition that varies significantly depending on the part of the plant used (Ozliman et al. 2021). The results also indicate that the extraction technique impacts the constituent composition, with trans dihydrocarvone (4.87%) being notably higher in steam‐distilled oils, while limonene, carvone, and dillapiole are more concentrated in oils extracted by super‐heated steam distillation.

### 3.4 Antioxidant Activity

The antioxidant activity of *A. graveolens* essential oil, assessed using various methods such as DPPH free radical scavenging activity, reducing power ability, and hydrogen peroxide free radical scavenging activity, demonstrates significant variations based on the extraction method used, as shown in Table 4. Notably, the SHSE method consistently results in the highest percentage of free radical scavenging activity (FRSA) across all three assays. This high FRSA is attributed to the essential oil's ability to donate hydrogen, effectively neutralizing oxidative stress by reducing the violet color of DPPH, as outlined by (Tsvetkov et al. 2019). These findings underscore the enhanced antioxidant potential of the EO extracted via SHSE, indicating superior efficacy in mitigating oxidative stress and promoting health benefits. Specifically, the EO from SHSE showed the highest DPPH scavenging activity at 87.48%. This aligns with previous studies that attribute the antioxidant properties of dill seed EO to its polar components, with monoterpenes like carvone and myrcene playing crucial roles. It has been observed that EO rich in monoterpenes displays greater antioxidant activity, with *S. carvone*, a key component of dill EO, known for its potent antioxidant effects, surpassing even α‐tocopherol in activity (Kaur et al. 2019). The total antioxidant content of dill seed EOs, particularly when extracted by SHSE, reached a peak of 163.06 mg/L of gallic acid equivalent. Components such as carvone, limonene, and carvone oxime contribute to moderate FRAP scavenging activity, with increased concentrations enhancing the FRAP activity percentage. Furthermore, monoterpenes are noted for their effective preservation actions. The highest FRSA was recorded by the DPPH assay at 87.48%, slightly below the standard BHT, while the lowest activity was observed in the hydrogen peroxide assay at 68.83% for EO extracted by hydro distillation. Generally, the standard antioxidants exhibit higher potential than the essential oil, yet the presence of compounds like dillapiole and anethol in the EO may enhance its antioxidant activity (Singh et al. 2005).

**TABLE 4:** Antioxidant activity of *Anethum graveolens* essential oils by DPPH, ferric reducing power assay, and hydrogen peroxide assay.

Extraction method	DPPH free radical scavenging activity (%)	Total antioxidant content/FRAP^A^	H_2_O_2_ scavenging activity (%)
Hydro‐distillation	78.23 ± 0.39^e^	109.56 ± 0.54^d^	68.83 ± 0.28^e^
Steam distillation	83.11 ± 0.28^d^	117.28 ± 0.35^c^	70.46 ± 0.14^d^
Supercritical carbon dioxide extraction	85.12 ± 0.18^c^	131.52 ± 0.26^b^	74.12 ± 0.29^c^
Superheated steam distillation	87.48 ± 0.12^b^	163.06 ± 0.56^a^	76.43 ± 0.22^b^
BHT^B^	97.54 ± 0.56^a^	—	—
Gallic acid^C^	—	—	85.48 ± 0.68^a^

*Note:* Values are mean ± SD of three separate determinations. Different letters in superscript represent significant differences among *Anethum graveolens* essential oils extracted by different extraction methods.

Abbreviations: BHT = butylated hydroxytoluene; DPPH = 2,2‐diphenyl‐1‐picrylhydrazyl; FRAP = ferric reducing antioxidant power.

^A^ Total antioxidant contents/FRAP (mg/L of essential oil, measured as gallic acid equivalent).

^B^ Positive control for DPPH free radical scavenging activity (BHT;100 ppm).

^C^ Positive control for H_2_O_2_ scavenging activity (Gallic acid; 100 ppm).

## 4 Conclusions

This research was done to extract essential oil from *A. graveolens* seeds using advanced techniques such as SHSE, SCF‐CO_2_, and conventional methods including HD and SD. The study assessed the impact of extraction techniques on EO yield, biological activities, and chemical profiling. Results demonstrated that SHSE significantly influenced the yield and biological properties of the essential oil. It was the EO extracted by SHSE that was obtained with the highest yield and that exhibited noteworthy antimicrobial and antioxidant potential. D‐carvone, d‐limonene, and dillapiole were the major components shown by the GC–MS of essential oil, likely responsible for the observed antioxidant and antimicrobial activities. Consequently, these findings suggest that superheated steam extraction is the optimal technique for extracting essential oils, offering increased yields and enhanced biological activities.

## Data Availability

The authors have nothing to report.
